# Role of Bacterial and Viral Pathogens in Gastric Carcinogenesis

**DOI:** 10.3390/cancers13081878

**Published:** 2021-04-14

**Authors:** Manikandan Palrasu, Elena Zaika, Wael El-Rifai, Jianwen Que, Alexander I. Zaika

**Affiliations:** 1 Department of Surgery, University of Miami, Miami, FL 33136, USA; mxp1405@med.miami.edu (M.P.); exz99@med.miami.edu (E.Z.); welrifai@med.miami.edu (W.E.-R.); 2 Department of Veterans Affairs, Miami VA Healthcare System, Miami, FL 33136, USA; 3 Department of Medicine, Columbia University Medical Center, New York, NY 10032, USA; jq2240@cumc2.columbia.edu

**Keywords:** gastric tumor, gastric pathogens, *H. pylori*, EBV, p53, p14ARF

## Abstract

**Simple Summary:**

Stomach cancer is one of the most common cancers in the world, with over one million new cases diagnosed in 2020. Despite recent advances in cancer treatments, gastric cancer remains a serious clinical problem. This disease is tightly linked to gastric infections with *Helicobacter pylori* bacterium, Epstein–Barr virus, and some other less known pathogens. Here, we discuss how gastric pathogens induce tumorigenic changes in the stomach.

**Abstract:**

Gastric cancer (GC) is one of the deadliest malignancies worldwide. In contrast to many other tumor types, gastric carcinogenesis is tightly linked to infectious events. Infections with *Helicobacter pylori* (*H. pylori*) bacterium and Epstein–Barr virus (EBV) are the two most investigated risk factors for GC. These pathogens infect more than half of the world’s population. Fortunately, only a small fraction of infected individuals develops GC, suggesting high complexity of tumorigenic processes in the human stomach. Recent studies suggest that the multifaceted interplay between microbial, environmental, and host genetic factors underlies gastric tumorigenesis. Many aspects of these interactions still remain unclear. In this review, we update on recent discoveries, focusing on the roles of various gastric pathogens and gastric microbiome in tumorigenesis.

## 1. Introduction

Approximately 13–15% of human cancers worldwide can be attributed to infectious agents [1]. One demonstrative example is gastric cancer (GC), which is strongly associated with infections caused by *Helicobacter pylori* (*H. pylori)* bacteria and other pathogens. Despite all efforts, gastric cancer remains a serious clinical problem. Over seven hundred thousands of deaths related to GC have been reported in 2020, ranking the fourth most-deadliest tumor in the World [2]. The incidence of GC is characterized by complex dynamics and geographical variation. Its occurrence slowly declines in North America and most Western European countries, but its burden remains very high in Asia, Latin America, and Eastern Europe [3]. Multiple histological and anatomical classifications of GC have been proposed over time. For more than half a century, the characterization of GC was largely based on Lauren’s criteria, in which GC was divided into intestinal, diffuse and, undetermined types [4,5]. In 2010, the World Health Organization (WHO) expanded this classification by identifying papillary, tubular, mucinous and poorly cohesive (including signet ring cell carcinoma and other variants), and unusual histological variants [6].

Another approach to GC classification is based on the molecular profiling using gene expression and DNA sequencing analyses. A comprehensive study by the Cancer Genome Atlas consortium (TCGA) proposed four molecular subtypes of GC: (1) tumors positive for Epstein–Barr virus (EBV), (2) microsatellite unstable tumors (MSI), (3) genomically stable tumors (GS), and (4) tumors with chromosomal instability (CIN) [7]. More clinically relevant molecular classification has been presented by the Asian Cancer Research Group (ACRG). This study used gene expression data to describe molecular subtypes linked to distinct patterns of molecular alterations and disease progression and prognosis. Based on these criteria, GC was separated into four groups: MSI-high, microsatellite-stable/p53 inactive (MSS/TP53−), microsatellite-stable/p53 active (MSS/TP53+), and microsatellite-stable/epithelial-to-mesenchymal transition (MSS/EMT) subtypes [8]. Additional classifications have also been proposed [9].

In this review, we discuss various bacterial and viral pathogens associated with tumorigenic alterations in the stomach.

## 2. *H. pylori* and Gastric Cancer

*H. pylori* is a spiral-shaped gram-negative microaerophilic bacterium that, in the process of evolution, adapted to survive and thrive in the human stomach. Since the seminal discovery of *H. pylori* and its role in gastritis and peptic ulcer disease by Robin Warren and Barry Marshall [10], studies of this pathogen have been continuing for more than three decades. Among many important findings during this period of time, discovery of the relationship between *H. pylori* and noncardia gastric cancer, and the characterization of gastric tumorigenesis as a stepwise inflammatory process, initiated by *H. pylori*, played key roles [11]. The stepwise model that emphasizes the role of chronic inflammation and consecutive pathological changes has been proposed by Dr. Pelayo Correa, and has stood the test of time [12]. According to this model, intestinal-type GC is the end result of lengthy progressive changes in the gastric mucosa that start with chronic gastritis, followed by atrophic gastritis, intestinal metaplasia (IM), dysplasia and invasive tumor. In 1994, *H. pylori* was recognized as a type I carcinogen by the International Agency for Research on Cancer [13]. The clinicopathological role of *H. pylori* was further highlighted by studies showing that *H. pylori* eradication reduces gastric inflammation and decreases the risk of premalignant and malignant lesions in the stomach [14]. Several effective anti–*H. pylori* treatment regiments have been developed and successfully used in clinic [15,16,17].

Besides IM, another type of metaplasia, called spasmolytic polypeptide-expressing metaplasia (SPEM), is also associated with chronic *H. pylori* infection and gastric adenocarcinoma [18]. It develops as a results of transdifferentiation of chief cells following persistent stomach injury and loss of parietal cells in the gastric oxyntic mucosa [18,19].

**Figure 1:**
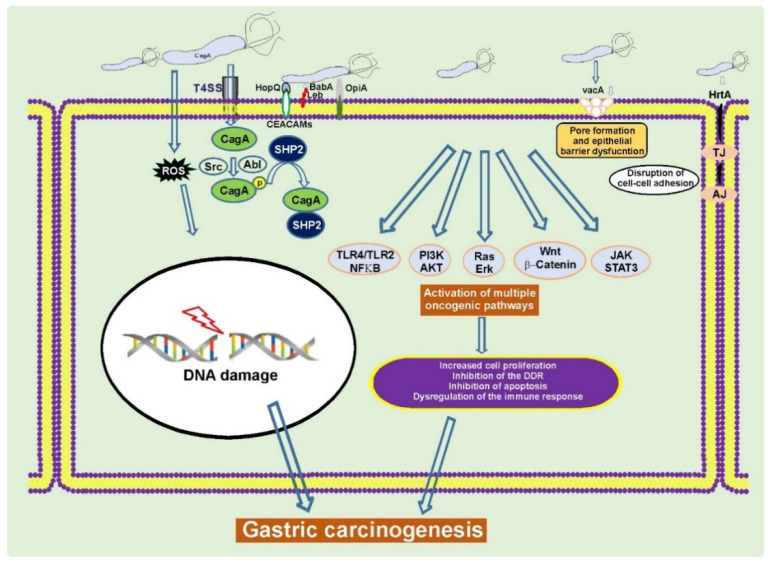
*H. pylori* alters cellular homeostasis during infection. *H. pylori* colonization of the human stomach is responsible for aberrant activation of multiple oncogenic pathways, induction of DNA damage, disruption of the epithelial barrier, and modulation of the host immune response. CagA, VacA, and other virulence factors play a key role in these processes [22,25,26].

*H. pylori* typically infects humans at an early age, leading to decades-long chronic infection and mucosal injury that may progress to GC at older age. *H. pylori* is responsible for almost 90% of all noncardia gastric cancers [11,20]. Although infection with *H. pylori* is very common worldwide, only a small fraction of infected individuals develops GC, indicating complexity of tumorigenic interactions between bacteria and host cells. Among *H. pylori* virulence factors cytotoxin-associated gene A (CagA) protein and vacuolating cytotoxin A (VacA) are the most studied determinants associated with gastric carcinogenesis (Figure 1) [21,22,23,24].

### 2.1. The cag Pathogenicity Island (cag PAI) and CagA Protein

The *cagA* gene, which encodes CagA protein, is located at the 3′ end of the *cag* pathogenicity island, a 40-kilobase bacterial genomic DNA fragment that is thought to be acquired by horizontal transfer of genetic material. The products of the *cag* PAI form highly organized type IV secretion system (T4SS) pili that functions as a sophisticated molecular machine delivering CagA inside gastric epithelial cells. There are also evidences that bacterial lipopolysaccharides, peptidoglycans, and DNA can be delivered by the T4SS [22,26,27,28,29]. After translocation, CagA is phosphorylated by host tyrosine kinases belonging to the SRC and ABL families at the EPIYA (Glu-Pro-Ile-Tyr-Ala) repeatable motifs located at the carboxy-terminal end of the CagA molecule. The EPIYA motifs are responsible for binding of CagA to multiple host proteins and dysregulation of their functions. Currently, four distinct EPIYA types (-A, -B, -C, and -D) have been identified based on surrounding amino acid sequences. The EPIYA motifs are commonly assembled in the A-B-C(D) arrangements, where the EPIYA-C and rarely EPIYA-D fragments can be present in multiple copies. *H. pylori* strains carrying the EPIYA-C and EPIYA-D motifs have different geographical distribution. The EPIYA-C motif is typically found outside East Asia, whereas the East Asian strains predominantly carry the EPIYA-D motif [30].

This phenomenon has clinicopathological significance. Systematic review and meta-analysis of published research have shown that the presence of EPIYA-D and multiple EPIYA-C motifs are significantly associated with an increased risk of gastric cancer in the United States/Europe and Asia [31,32].

In addition to the EPIYA motif, the C-terminus of the CagA protein contains another repeatable sequence named the CagA-multimerization motif (CM) [33]. The CM motif comprises 16 amino acid residues and is responsible for homodimerization of CagA and interaction with PAR1b/MARK kinase, playing a critical role in the epithelial cell polarity. [33]. East Asian CagA usually has a single copy of East Asian type of the CM motif, while Western CagA retains multiple copies of Western type of the CM motifs. The polymorphism of the CM and EPIYA motifs explains differences in molecular weight of CagA protein that can vary from 120 to 145 kDa between *H. pylori* variants [33].

Based on current understanding, CagA is the most significant single factor defining gastric tumorigenesis. Multiple human studies have found considerable associations between infections with CagA-positive *H. pylori* bacteria and an increased risk of gastric cancer [21,34,35,36]. There are also multiple experimental evidences showing that CagA functions as an oncoprotein. CagA transgenic mice, in which effects of other virulence factors were excluded, developed gastric epithelial hyperplasia and hematopoietic and gastrointestinal malignancies, including gastric adenocarcinoma [37]. Similarly, transgenic expression of CagA in zebrafish causes intestinal epithelial hyperplasia and, in combination with loss of p53, produces intestinal small cell carcinomas and adenocarcinomas [38]. CagA also enhances growth and invasion of tumors generated by expression of oncogenic Ras in Drosophila [39].

**Figure 2:**
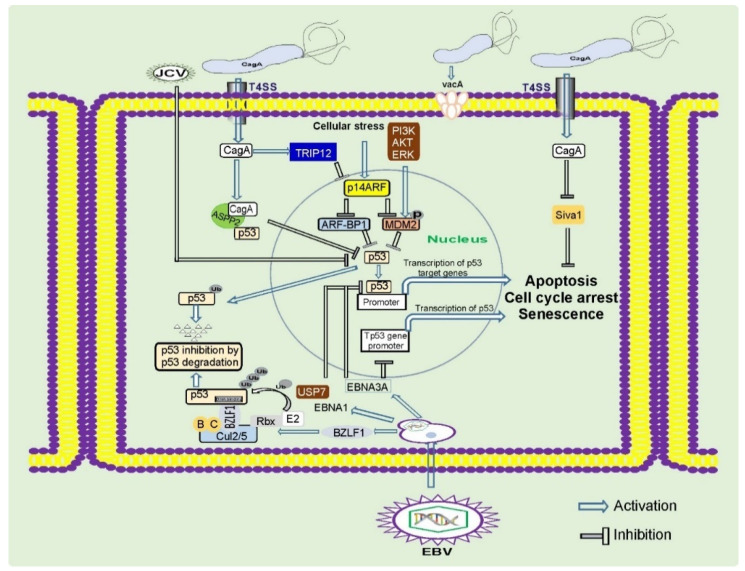
Regulation of tumor suppressor proteins by gastric pathogens. Gastric pathogens: *H. pylori* and oncogenic viruses inhibit key tumor suppressors proteins p53, p14ARF, and others. These events result in inhibition of the DNA damage and oncogenic stress responses, two key mechanisms important for prevention of gastric carcinogenesis.

Oncogenic pursuit of CagA is mediated by aberrant activation of multiple signaling cascades that are known to be altered in gastric cancer (RAS/ERK, WNT/β-catenin, JAK/STAT, PI3K/AKT, and others) and inhibition of tumor suppressors. CagA is the first bacterial protein that has been shown to induce degradation of p53 tumor suppressor, activating the PI3K/AKT/MDM2/ARF-BP1 and ERK/MDM2-pathways [40,41] (Figure 2). Previously, only viral proteins, such as HPV E6, were known to degrade p53 [30]. CagA is responsible for altering expression of N-terminally truncated p53 isoforms: ∆133p53 and ∆160p53 [42]. Interestingly, the dysregulation of p53 occurs in a strain-specific manner, with tumorigenic *H. pylori* strains having a stronger ability to affect p53 [40,43]. Tumorigenic *H. pylori* strains also decrease activity of other tumor suppressors: p14ARF, SIVA1, and p27(KIP1) [43,44,45,46].

Interaction of *H. pylori* with gastric cells increases the levels of reactive oxygen and nitrogen species and induces oxidative stress and DNA damage in a CagA-dependent and -independent manner [47,48,49,50]. Although the entire spectrum of *H. pylori*–induced DNA damage is currently unknown, the formation of oxidized nitrated DNA lesions and single- and double-strand DNA breaks has been shown. Double-strand breaks in DNA are particularly detrimental, as these lesions are extremely difficult to repair resulting in highly cytotoxic and mutagenic effects [47,49,51,52,53,54,55,56]. *H. pylori* can also induce damage of mitochondrial DNA likely contributing to cellular senescence and gastric cancer initiation [57].

Induction of DNA damage by *H. pylori* is exacerbated by inhibition of p53 and multiple DNA repair pathways that are important for proper activation of the DNA damage response [40,42,49,52,58,59,60,61].

CagA is known to function as an anti-apoptotic protein. Multiple prosurvival factors and pathways have been shown to be induced by CagA, and among them are kinases AKT and ERK; antiapoptotic members of the B-cell lymphoma 2 (BCL-2) protein family MCL-1, BCL-2, and BCL-Xl; and others [62,63,64,65]. CagA is responsible for the suppression of proapoptotic factors such as SIVA1, BIM, and BAD; downregulation of autophagy; and induction of inflammation [46,62]. Human infections with CagA-positive *H. pylori* strains are characterized by strong inflammation and severe damage of gastric tissues [66,67,68,69,70].

It has been reported that CagA protein has a profound impact on various cellular functions, including epithelial cell barrier, cell polarity, proliferation, apoptosis, EMT, autophagy, miRNA biogenesis, inflammatory and DNA damage responses, and others. It affects activities of multiple kinases and cell signaling pathways. A partial list includes the following: EGFR, c-MET, SRC, cABL, CSC, aPKC, PAR1, PI3K, AKT, FAK, GSK-3, JAK, PAK, MAP, MDM2, p53, p14ARF, p27, RAS, β-catenin, NFκB, and multiple NFκB-related pathways [43,44,46,71,72,73,74,75,76,77,78]. It is not completely clear how one bacterial protein produces so pleiotropic effect. One plausible explanation is that CagA acts as a scaffolding protein that interacts with a large number of the host regulatory proteins, tethering them into aberrant enzymatic complexes and altering their normal functions [79].

### 2.2. VacA and Other Virulence Factors

VacA toxin is another virulence factor that plays a major role in tumorigenesis, associated with *H. pylori* infection. Its name originates from the ability to cause cell vacuolation in cultured eukaryotic cells. VacA has been classified as a pore-forming toxin. Although many toxins can form pores, the amino acid sequence of VacA is not closely resembled sequences of other known bacterial toxins [22,80,81]. The biosynthesis of VacA includes several sequential steps. Following protein translation, the VacA precursor undergoes complex proteolytic cleavage that produces 88 kDa active toxin that either secreted into the extracellular space or retained on the bacterial surface. The secreted VacA protein binds to target cellular membranes, forming an anion-selective membrane channel [82,83].

Multiple functions have been found to be associated with VacA activity, including disruption of the gastric epithelial barrier, interference with antigen presentation, suppression of autophagy and phagocytosis, inhibition of T cells and B cells that are thought to help bacteria to establish persistent infection [84,85,86,87,88]. The ability of VacA to inhibit autophagy and lysosomal degradation facilitates the accumulation of oncogenic protein CagA in gastric epithelial cells [89].

There are considerable variations in VacA sequences. Three main regions of diversity have been recognized in VacA: the signal sequence region (or “s”), the intermediate region (or “i”), and the middle region (or “m”). Based on sequence heterogeneity, the s region was subdivided into s1 (further subdivided into s1a, s1b, and s1c) and s2 types, the i region was subdivided into i1 and i2 types, and the m region was subdivided into m1 and m2 (further subdivided into m2a and m2b) types [85,90]. The incidence of GC has been found to be higher in populations infected with *H. pylori* variants containing type s1/i1/m1 of *vacA*, compared to populations infected with *H. pylori* type s2/i2/m2 of *vacA* [36,84,85]. Bacterial strains carrying type s1 and m1 *vacA* alleles have been associated with epithelial damage, increased gastric inflammation, and duodenal ulceration [91,92,93].

Besides CagA and VacA, *H. pylori* expresses a number of other cancer-associated virulence determinants. Outer-membrane proteins (OMPs) are among them. These proteins are important for bacterial adherence, colonization, survival, and persistence [94]. These factors also promote gastric diseases by affecting the signaling pathways in the host cells, enhancing activity of the T4SS, and altering immune responses [94]. *H. pylori* expresses a large repertoire of OMPs divided into five major families based on their sequence similarities [95]. The largest and the most studied family is the Family 1, which comprises the Hop (for *H. pylori* OMP) and Hor (for Hop related) proteins. The two most studied *H. pylori* adhesins in the Hop subgroup are BabA(HopS) and SabA(HopP), which have been originally identified to interact with the fucosylated-Lewis B (Le^B^) and the sialylated-Lewis X (sLe^X^) blood group antigens, respectively, mediating binding of *H. pylori* to extracellular matrix and gastric epithelial cells [96,97]. BabA potentiates activity of the T4SS [98] and is involved in induction of double-strand breaks in host cells [49]. SabA increases the colonization density and inflammation in human stomach [97,99]. Several studies analyzed associations of BabA and SabA expression with clinical outcome. The BabA status of infecting bacteria has been found to be associated with the presence of intestinal metaplasia, gastric adenocarcinoma, and MALT (Mucosa-Associated Lymphoid Tissue) lymphoma [96,100,101,102,103,104]. Similarly, the SabA status was correlated with an increased risk of premalignant lesions and gastric cancer [105,106]; however, some studies produced contradictory results [99,107].

Other OMPs, such as OipA(HopH), HopQ, and HomB, have also been implicated in gastric tumorigenesis [102,108,109,110,111,112,113,114,115]. Further studies are needed to better characterize properties of OMPs and their roles in gastric tumorigenesis.

## 3. Gastric Microbiota

The stomach is not a sterile organ, despite its high acidity. It is populated by complex gastric microbial communities that affect tumorigenic processes and are important for the maintenance of human health.

The composition of normal gastric microbiota is diverse and highly dynamic with the most abundant phyla: *Proteobacteria*, *Firmicutes*, *Bacteroidetes*, *Fusobacteria*, *Actinobacteria*, and others [116,117]. On the other hand, *H. pylori* has been found to be the most prevalent bacteria in the stomach of *H. pylori*-infected individuals [116,118]. *H. pylori* can induce profound changes in the composition of gastric microbiota [117,119,120,121,122,123,124,125]. Analyses of gastric microbiota in specific pathogen-free (SPF) mice revealed that *H. pylori* infection decreases abundance of normal gastric flora, such as *Lactobacilli*, and increases the presence of *Clostridia*, *Ruminococcus* spp., *Eubacterium* spp., *Bacteroides*/*Prevotella* spp., and others [122]. Similar phenomenon was observed in Mongolian gerbils [124,125,126]. These alterations can be explained, at least in part, by physiological changes caused by persistent *H. pylori* infection [127]. Induction of chronic inflammation and suppression of acid production can facilitate growth of various non–*H. pylori* bacterial species [127,128,129]. Many aspects of these interactions still remain controversial. Some studies did not find significant differences in the microbial composition between *H. pylori*–positive and –negative individuals [116,130,131]. It is likely that multiple confounding factors, such as level and type of inflammation, drug treatment (such as treatment with proton pump inhibitors), and the presence of precancerous and cancerous lesions, have to be taken into consideration during analyses of gastric microbiota.

Phylogenetic diversity of the stomach microbiome is changed during progression from gastritis to intestinal metaplasia and GC in human patients [120,132,133,134]. *H. pylori* colonization of the human stomach is frequently decreased in patients with advanced premalignant and malignant lesions, while abundance of *Streptococcus*, *Lactobacillus*, *Veillonella*, *Clostridia*, and others is increased [132,133,134,135,136]. Decline in *Porphyromonas*, *Neisseria*, and *S. sinensis* species and concomitant increase in *Lactobacillus* *coleohominis* and *Lachnospiraceae* were found to correlate with progression from gastritis to GC [133]. Changes in the gastric microbiota were also observed after surgical treatment of GC patients [137].

Synergetic interactions of bacteria with *H. pylori* to promote gastric neoplasia have been convincingly demonstrated by using transgenic insulin–gastrin (INS–GAS) mice [138,139]. It was found that *H. pylori* infection causes less severe gastric lesions and delayed onset of gastric intraepithelial neoplasms (GINs) in germ-free INS–GAS mice compared to mice with complex gastric microbiota [140]. In another study, infection of INS–GAS mice with restricted Altered Schaedler flora (rASF), containing *Clostridium*, *Lactobacillus*, and *Bacteroides* species, was sufficient to develop gastric dysplasia [141]. Infection with *H. pylori* further accelerated the onset of gastric lesions in rASF-infected mice [142]. Notably, antimicrobial therapies delayed onset of GIN not only in INS–GAS mice infected with *H. pylori*, but also in animals without *H. pylori* infection, thus indicating that non–*H. pylori* bacteria, including those considered as commensals, may represent an additional GC risk, particularly in *H. pylori*–infected susceptible individuals. [140,142,143,144].

It is not completely clear how non–*H. pylori* microbiota synergizes with *H. pylori* to induce GC. One plausible explanation includes overgrowth of bacteria, converting nitrogen compounds into potentially carcinogenic N-nitroso compounds [129]. It was shown that reduction of gastric acidity causes growth of nitrate-reducing bacteria, which produce carcinogenic N-nitrosamine [128,145,146]. It is also possible that various non–*H. pylori* bacteria promote sustained inflammation that contributes to development of GC.

Interactions within the gastric microbiome are complex and may result in various outcomes. Colonization of C57BL/6 mice with the enterohepatic *Helicobacter* species, *H. bilis* or *H. muridarum*, before challenge with *H. pylori*, was found to reduce *H. pylori*–induced gastric injury [147,148]. Similarly, oral *Lactobacillus* strains were shown to suppress *H. pylori*– and *H. felis*–induced inflammation in both mice and gerbils [125,149,150,151,152]. Consistent with rodent models, certain *Lactobacilli* were also found to suppress *H. pylori* growth and gastric mucosal inflammation in human individuals [153,154].

One interesting aspect of complex biological interactions in the stomach is the influence of helminthiasis. Parasitic worms are known to be involved in the development of various human malignancies [155]. However, certain types of helminths can decrease the risk of GC [156,157]. Infection of mice with enteric helminth (*Heligmosomoides polygyrus)* has been found to attenuate progression of premalignant gastric lesions induced by *H. pylori* and *H. felis* [156,158]. It was also suggested that helminths may decrease the incidence of *H. pylori*-associated GC in certain world populations due to their immunomodulating effects [157,159].

## 4. EBV and Gastric Carcinogenesis

In addition to pathogenic bacteria, compelling evidences point to significant contribution of viral infections to gastric carcinogenesis [1,160,161,162]. EBV is the most characterized gastric oncogenic virus. EBV is a member of the herpes virus family (*Herpesviridae*) and one of the eight known herpesviruses infecting humans. EBV is classified as the human gamma herpesviruses 4 (HHV-4). It was discovered in cultured tumor cells derived from African Burkitt’s lymphoma in 1964 [163]. Successive studies revealed that EBV infects more than 90% of the world’s population [164]. Infection with EBV typically occurs at a young age. Virus is often transmitted through saliva, infecting oral epithelial cells and multiple types of immune cells. EBV infections are usually asymptomatic, but some patients develop the clinical syndrome of infectious mononucleosis that predominantly affects adolescents and young adults. The occurrence of EBV-associated gastritis has also been reported, especially in cases of co-infection with *H. pylori* [165].

The most severe consequence of EBV infection is the development of tumors. Infection with EBV is strongly associated with the various types of lymphomas, and non-lymphoid malignancies, such as leiomyosarcoma and gastric and nasopharyngeal carcinomas. EBV contributes to approximately 1.5% of all cases of human cancers worldwide. Similar to *H. pylori*, it has been classified as a group I carcinogen by the International Agency for Research on Cancer [1,166,167,168]. Interestingly, EBV-associated GC (EBVaGC) has been found to be correlated with a higher survival rate than other GC subtypes [169]; but some studies produced contradictory results [170]. A number of antiviral treatments against EBV have been found to efficiently inhibit viral replication in laboratory testings [171,172]. However, they have had limited success in clinic and are not currently approved for treatment of EBV infection [173]. At the same time, promising results were reported in treatment of EBVaGC based on its distinct pathological characteristics [174].

Based on differences in sequences of the EBNA latency genes, EBV strains have been classified as type 1 (T1) and type 2 (T2) [175]. T1 EBV infections are more common worldwide and primarily found in Europe, Asia, North and South America, whereas T2 strains are prevalent in Africa and New Guinea [176,177]. The role of specific EBV types in the etiology of different cancers is not clear, however accumulating data suggest that additional cofactors (co-infections, comorbidities, etc.) significantly contribute to development of EBV-associated malignances [178,179,180,181].

The association of EBV with lymphoepithelial carcinoma of the stomach was initially reported by Burke et al. the in 1990 [182]. Two years later, Shibata and Weiss, detected EBV genetic material in 16% of gastric adenocarcinomas. The EBV has been specifically found within GC cells and adjusted dysplastic epithelium, but not in surrounding lymphocytes and normal gastric mucosa [183]. EBV is primarily localized in the proximal stomach and manifest itself as adenocarcinoma or as rare lymphoepithelioma-like carcinoma. The comprehensive genomic analysis conducted by the TCGA revealed that approximately 9% of human gastric cancers are positive for EBV. Multiple molecular abnormalities have been detected in EBVaGC: extensive DNA hypermethylation; high-frequency mutations of PIK3CA, ARID1A, and BCOR; and amplification of JAK2, PD-L1, and PD-L2 [7,184]. A closely followed study by the ACRG found 6.5% of EPV-positive GC patients [8,185]. An even higher positivity rate has been reported, when EBV was detected by RNA-Seq instead of traditional EBER1/2 *in situ* hybridization [186].

The EBV entry into epithelial cells is relatively well-studied. It involves multiple interactions between viral and host proteins. Among host proteins, ephrin receptor A2, integrins (αVβ5, αVβ6, and αVβ8), neuropilin 1, complement receptor type 2 (*CR2*), and nonmuscle myosin heavy chain IIA help virus entry [187]. It is not completely clear how EBV infects gastric epithelium, owing to the hostile acidic environment of the stomach. Most plausibly, EBV is delivered by infected B lymphocytes, attracted to the stomach by inflammatory processes that precede gastric tumor development [188]. It was found that membrane vesicular products secreted by epithelial cells can activate virus in latent EBV-infected B lymphocytes, resulting in virus production and infection [189]. Another possibility is that EBV is delivered in contaminated saliva, which is constantly ingested, and, in certain circumstances, the virus may survive in the harsh environment of the stomach and infect gastric mucosal cells [167].

One prominent feature of EBV is its ability to establish chronic infections alternating lytic and latent virus cycles. Following the virus entry into host cells, double-stranded viral DNA is maintained as a multiple-copy episome, establishing the base for latent infection that is characterized by remarkable variation and plasticity in viral transcription and replication. Depending on the pattern of viral gene expression, primary EBV infection can be categorized into three latency types (latency types I, II, and III). The latent pattern of EBVaGC corresponds to the unique latency I/II with expression of EBV-determined nuclear antigen 1 (EBNA1), noncoding RNAs (EBER1, EBER2), and BamHI-A rightward transcripts (BART miRNAs) that is characteristic for the latency type I. In addition, latent membrane protein 2 (LMP2A), which is characteristic for latency type II, is expressed in approximately 50% of EBVaGCs [190,191]. Expression of both latent and lytic genes play an important role in gastric carcinogenesis [192].

Several latency factors have been shown to have oncogenic properties. Among them is EBV-encoded latent membrane protein 2A (LMP2A) that activates the PI3K/AKT proliferation pathway, increases survival of infected cells via upregulation of survivin gene expression, inhibits TGFβ1-induced apoptosis, and promotes cellular migration through targeting the Notch signaling pathway [193,194,195]. LMP2A is also responsible for activation of DNA methyltransferase 1 (DNMT1) via STAT3 pathway, leading to the promoter methylation of PTEN tumor suppressor [196].

Another latency factor, EBNA 1 protein that is responsible for viral DNA replication and EBV persistence, induces protein degradation of promyelocytic leukemia (PML) tumor suppressor, inhibiting the formation of nuclear bodies (PML NBs). This inhibition results in impairment of the DNA damage response and increase in cell survival [197].

Not only viral proteins are responsible for tumorigenic alterations. EBV produces multiple viral noncoding RNAs (ncRNAs) during the acute and latent stages of infection that regulate expression of viral and host genes. Among them are EBV-encoded RNAs (EBERs), BamHI-A rightward transcripts (BARTs), viral small nucleolar RNA1 (v-snoRNA1), and viral microRNAs (miRNAs). They play a critical role in host cell proliferation, survival, apoptosis, immune escape, and regulation of host ncRNAs [198]. The list of newly discovered EBV ncRNAs keeps growing.

EBV causes multiple epigenetic abnormalities in host cells. It has been found that Tet Methylcytosine Dioxygenase 2 (TET2), a regulator of DNA methylation, is downregulated during EBV infection, contributing to an abnormal DNA methylation profile [199]. As a result, many tumor-suppressor genes, such as *p16*, *p14*, *APC*, and *TP73*, become methylated [200,201,202,203,204,205].

Similar to *H. pylori*, EBV inhibits activity of p53 tumor suppressor. Interestingly, multiple viral proteins are involved in this process. EBNA1 and EBNA3C repress p53-dependent transcription and augment its ubiquitination and degradation [206]. The immediate-early protein BZLF1 (BamHI Z fragment leftward open reading frame 1) also inhibits p53-dependent transcription [207,208]. This protein functions as an adaptor component of the ECS ubiquitin ligase complex (Elongin B/C-Cul2/5-SOCS-box protein) that facilitates p53 degradation [209] (Figure 2). Direct viral inhibition of p53 might explain why EBVaGC rarely harbors mutations in the *TP53* gene [7].

## 5. Other Carcinogenic Viruses

In addition to EBV, a number of viruses have been found to contribute to tumorigenic alterations in the stomach. Among them is human polyomavirus 2, commonly referred as the John Cunningham virus (JCV). JCV is a small DNA virus and etiological agent of progressive multifocal leukoencephalopathy (PML), a debilitating and frequently fatal central nervous system disease. JCV infection typically occurs during childhood and persists for the lifetime of the host [210,211]. Immune system of healthy individuals prevents JCV replication suppressing the virus and keeping it in the latent state, but JCV can be reactivated as a result of various immunodeficiencies.

Several studies reported the presence of JCV in gastric, esophageal, and colorectal cancer tissues [212,213,214,215]. Expression of JCV T-antigen (T-Ag) in gastric tumor tissues was found to be in the range of 26–86% [214,215,216]. It was shown the correlation of JCV infection with activation of β-catenin, absence of p53 mutations, allelic losses and aberrant methylation of multiple genes, including tumor suppressors *p14* and *p16* [214,215,216,217]. The mechanism of JCV-induced gastric tumorigenesis is not well understood, but is likely mediated by large and small T-antigens that can inhibit retinoblastoma protein (pRb) and p53 tumor suppressor, activate β-catenin, and dysregulate cell cycle in infected cells [218,219,220].

Another virus that promotes gastric tumorigenesis is the human cytomegalovirus (HCMV), also termed human herpesvirus 5 (HHV-5). It is a member of the *Herpesviridae* family that, similar to EBV, is widely distributed around the World, with a prevalence of about 80% in industrialized countries and almost 100% in developing countries [221]. After initial infection, HCMV commonly remains in the latent state, but it can be activated in immunocompromised individuals. Several studies reported that HCMV infection is associated with an increased risk of GC [222,223,224]. The HCMV load was found to be significantly higher in GC epithelium compared to non-tumorous tissues. HCMV infection was also correlated with early onset of GC, lymphatic metastasis, and inhibition of negative regulator of the Wnt signaling pathway CTNNBIP1 [223,225,226].

Chronic infections with hepatitis B (HBV) or C (HCV) viruses are known risk factors for hepatocellular carcinoma. However, discovery of viral antigens and DNA in gastric tissues suggested a possible involvement of these viruses in gastric tumorigenesis. Histological examinations have shown that viral markers, HBV X protein (HBx) and core antibody (HBcAb), were expressed at higher levels in gastric tumor tissues than in normal counterparts [227]. Some case-control and population-based studies reported correlations between gastric precancerous and cancerous lesions and the presence of HBV surface antigen (HBsAg) or HCV antibody (HCV Ab) [228,229,230,231,232]. A recent meta-analytical study was consistent with these findings and reported association between HBV infection and an increased risk of GC [227]. Another recent large-scale study found that eradication of HCV reduces the risk of gastric cancer, particularly among younger individuals [233].

Infections with human retroviruses HIV (human immunodeficiency virus) or HTLV (human T-cell lymphotropic virus) increase the risk of developing various tumors, and can also indirectly affect gastric tumorigenesis [234,235]. HIV infection, if left untreated, leads to acquired immunodeficiency syndrome, a disease characterized by immune suppression and loss of immune-mediated control against diverse opportunistic pathogens. The analyses of HIV-positive patients found a significantly higher load of EBV DNA than in uninfected individuals [236,237]. Sixty percent of HIV-infected patients were positive for antibodies to EBV early antigen (EA) compared to 12% of uninfected individuals [237]. GC precursor lesions, including intestinal metaplasia and atrophic gastritis, were also common in patients infected with HIV [234]. In contrast to HIV, a large population-based study of HTLV-1-infected subjects found a reduced risk of GC development [238]. A recent meta-analysis of epidemiological studies further confirmed this correlation [235].

Currently, involvement of human papillomaviruses (HPV) in gastric tumorigenesis remains to be controversial and requires further investigation. While some studies suggested a potential link between HPV infection and gastric tumor, and also reported the presence of high-risk carcinogenic HPVs in GC and gastritis tissues [239,240], other studies did not found such associations [241,242].

## 6. Conclusions

**Figure 3:**
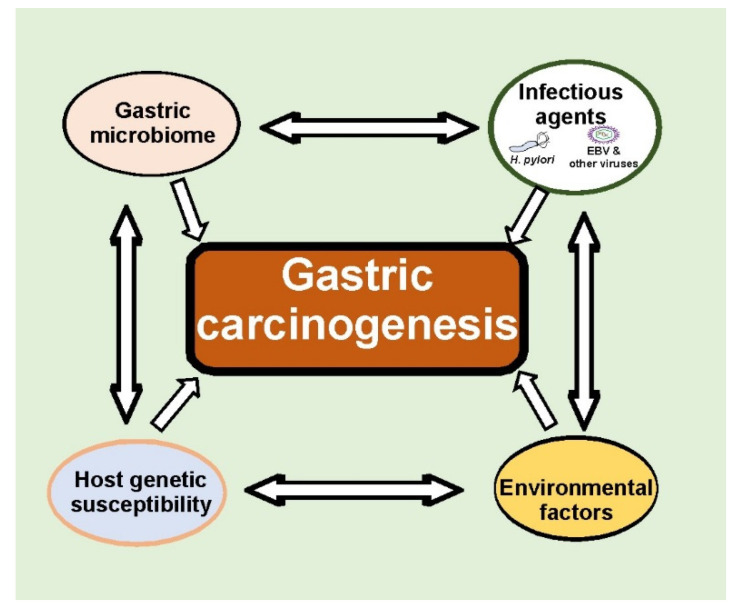
Multifaceted interactions between infectious agents, gastric microbiomes, and host genetic and environmental factors define tumorigenic processes in the stomach. Many aspects of these interactions remain currently unknown.

Gastric cancer is a heterogeneous disease developed as a result of multifactorial interactions between infectious agents, the gastric microbiome, and host genetic and environmental factors (Figure 3). Significant progress has been made on the way toward appreciation complexity of host–pathogen interactions. These findings open up new exciting avenues for future research that will certainly lead to a better understanding of gastric tumorigenesis and novel, more effective GC therapies.

## Data Availability

Not applicable.
